# Comparative Analysis of Microbial Species and Multidrug Resistance Patterns Associated with Lower Urinary Tract Infections in Preterm and Full-Term Births

**DOI:** 10.3390/microorganisms12010139

**Published:** 2024-01-10

**Authors:** Felix Bratosin, Roxana Folescu, Pavel Krupyshev, Zoran Laurentiu Popa, Cosmin Citu, Adrian Ratiu, Ovidiu Rosca, Adrian Cosmin Ilie

**Affiliations:** 1 Department of Infectious Diseases, “Victor Babes” University of Medicine and Pharmacy Timisoara, Eftimie Murgu Square 2, 300041 Timisoara, Romania; felix.bratosin@umft.ro (F.B.); rosca.ovidiu@umft.ro (O.R.); 2 Doctoral School, “Victor Babes” University of Medicine and Pharmacy Timisoara, Eftimie Murgu Square 2, 300041 Timisoara, Romania; 3 Methodological and Infectious Diseases Research Center, “Victor Babes” University of Medicine and Pharmacy Timisoara, Eftimie Murgu Square 2, 300041 Timisoara, Romania; 4 Department of Family Medicine, “Victor Babes” University of Medicine and Pharmacy Timisoara, Eftimie Murgu Square 2, 300041 Timisoara, Romania; folescu.roxana@umft.ro; 5 Faculty of General Medicine, I.M. Sechenov First Moscow State Medical University, Bolshaya Pirogovskaya Ulitsa 2, 119435 Moscow, Russia; krupyshev1002@gmail.com; 6 Department of Obstetrics and Gynecology, “Victor Babes” University of Medicine and Pharmacy Timisoara, Eftimie Murgu Square 2, 300041 Timisoara, Romania; citu.ioan@umft.ro (C.C.); ratiu.adrian@umft.ro (A.R.); 7 Department III Functional Sciences, Division of Public Health and Management, “Victor Babes” University of Medicine and Pharmacy Timisoara, 300041 Timisoara, Romania; ilie.adrian@umft.ro

**Keywords:** urinary tract infections, microbial resistance, prematurity, case study

## Abstract

The rise of multidrug-resistant organisms has significantly complicated the clinical management of urinary tract infections (UTIs), particularly in the context of pregnancy. This study aimed to identify and analyze the significant differences in microbial species and multidrug resistance patterns associated with UTIs in preterm versus full-term births, determine the bacterial species significantly associated with preterm birth, and describe the antibiotic resistance patterns affecting pregnant women with UTIs. This case–control study was conducted in western Romania and focused on pregnant women with UTIs admitted from 2019 to 2023. Data were retrospectively collected from 308 patients with positive cultures. Statistical analyses, including the Chi-square test, Fisher’s exact test, and logistic regression models, were employed to compare the proportions of microbial species and resistance patterns between preterm (*n* = 126) and full-term (*n* = 182) birth groups and identify factors independently associated with preterm birth. The study found no significant differences in demographic or lifestyle factors between the groups. However, significant differences were observed in several infection and inflammation markers. The median white blood cell count was higher in the preterm group (12.3 vs. 9.1, *p* = 0.032), and the median C-reactive protein level was significantly higher in the preterm group (18 vs. 7, *p* < 0.001). The preterm group exhibited a higher incidence of multidrug-resistant organisms, notably ESBL-producing organisms (19.8% vs. 4.4%, *p* < 0.001) and carbapenem-resistant Enterobacteriaceae (4.8% with *p* = 0.003). Notably, the resistance to amoxicillin was significantly higher in the preterm group (20.6% vs. 6.6%, *p* < 0.001). Significant bacterial associations with preterm births included Group B *Streptococcus* (OR 2.5, *p* = 0.001) and *Enterobacter* spp. (OR 1.8, *p* = 0.022). The study confirmed significant differences in microbial species and multidrug resistance patterns between UTIs associated with preterm and full-term births. The higher prevalence of certain bacteria and increased resistance to commonly used antibiotics in the preterm group underscore the need for tailored antimicrobial therapies and robust microbial identification in managing UTIs during pregnancy.

## 1. Introduction

Preterm birth, defined as delivery before 37 gestational weeks, is a leading cause of neonatal and infant morbidity and mortality [1]. Urinary tract infections (UTIs) have been identified as a significant risk factor for preterm birth, particularly in the setting of Group B *Streptococcus* infections [2,3]. Studies have shown that women with a UTI, particularly those requiring an emergency department visit or hospitalization, face an increased risk of preterm and early term births [4,5]. This is a critical concern, as UTIs are one of the more common perinatal complications, affecting approximately 8% of pregnancies and representing a spectrum from asymptomatic bacteriuria to more severe infections like pyelonephritis [6].

UTIs represent a significant public health issue, affecting an estimated 150 million people globally each year [7], predominantly arising from uropathogenic Escherichia coli, accounting for approximately 80% of cases [8,9]. However, the microbial landscape of UTIs is complex and varied, influenced by a myriad of factors, including the host’s physiology, age, and underlying conditions [10]. The role of the gut, vaginal, and urinary microbiome is increasingly recognized in the pathogenesis of UTIs, where specific uropathogens residing in the gut contaminate the periurethral space, leading to urethral colonization and eventual ascension to the urinary bladder [11].

Traditional urine culture and microscopy techniques have long been the cornerstone of UTI diagnosis [12]. The rate of false-negative and sterile cultures in UTIs is a critical consideration for accurate diagnosis and treatment. Urine cultures, while standard for UTI diagnosis, are known to have a high frequency of both false-positive and false-negative results [13]. The diagnostic threshold itself is often debated, with true UTIs sometimes associated with uropathogen growth of less than 100,000 CFU/mL, challenging the conventional threshold of ≥100,000 CFU/mL traditionally used to indicate an infection [14]. Nevertheless, recent advances, particularly next-generation sequencing (NGS), have revealed a more diverse microbial environment than previously understood. NGS has proven more sensitive in identifying a wider array of urinary bacteria, offering a more nuanced understanding of the microbial diversity involved in UTIs compared with conventional culture methods [15].

The rise of multidrug-resistant organisms has complicated the clinical management of UTIs [7,16]. The evolving resistance patterns necessitate a continuous re-evaluation of empirical treatment strategies and highlight the importance of tailored antimicrobial therapies based on robust microbial identification and sensitivity testing [17]. Therefore, this study hypothesizes that significant differences exist in the microbial species and multidrug resistance patterns associated with UTIs in preterm versus full-term births. The objectives are to comparatively analyze these differences, determine the bacterial species that are significantly associated with preterm birth, and describe the antibiotic resistance patterns affecting pregnant women with UTIs.

## 2. Materials and Methods

### 2.1. Research Framework and Ethical Considerations

This case–control study took place in western Romania at the Obstetrics and Gynecology unit of the Clinical County Hospital, focusing on patients with UTIs during the pregnancy period, admitted from 2019 to 2023. The hospital’s institutional review board granted ethical approval, adhering to the Declaration of Helsinki’s guidelines, on 31 March 2023 (code E-1853). Data were retrospectively collected from patients’ paper and digital records, selecting only cases with positive cultures. Prior to data collection, all participants provided informed consent for personal and medical records being used in research studies. The confidentiality and privacy of patient data were strictly maintained.

### 2.2. Participant Selection and Definitions

Inclusion criteria for the study comprised: (1) adult pregnant women aged 18 years and older; (2) a confirmed diagnosis of lower UTI during pregnancy, evidenced by positive urine cultures; (3) clearly recorded gestational age at the time of UTI diagnosis, with infections categorized by trimester; (4) comprehensive medical records available for the study period, including details of UTI episodes, treatments, and outcomes; (5) documented microbial identification and sensitivity profiles from urine cultures; (6) informed consent provided by the participants for the use of their medical and personal records in the research.

Exclusion criteria included: (1) history of pregnancy complications known as risk factors for preterm birth (previous preterm birth, twin pregnancies, uterine and cervical abnormalities, history of preeclampsia, placental abnormalities, artificial fertilization, thrombophilia and other genetic abnormalities), (2) sterile urine cultures, (3) yeast infections, (4) complicated UTIs, and (5) incomplete medical records or lack of informed consent. Patients were further described by the gestational age when infection occurred. The first trimester of pregnancy is considered a gestational age of less than 14 weeks, the second trimester is considered as an age between 14 and 26 weeks of pregnancy, while the third trimester is considered as any age above the gestational age of 26 weeks.

### 2.3. Study Variables

The study variables encompassed demographic details, lifestyle factors, medical history, and laboratory results. Demographics included age and BMI, while lifestyle factors covered smoking and alcohol use during pregnancy. Medical history variables were parity, trimester of infection, previous UTI, hypertension, diabetes, anemia, asthma, among others. Laboratory data spanned blood tests and urine culture results, including WBC, lymphocytes, neutrophils, PLT, RBC, hemoglobin, CRP, creatinine, and urea. Microbial identification focused on various Gram-negative and Gram-positive bacteria. The study also investigated multidrug-resistant microorganisms and antibiotic resistance patterns such as extended-spectrum beta-lactamases (ESBL), methicillin-resistant Staphylococcus aureus (MRSA), vancomycin-resistant Enterococci (VRE), and carbapenem-resistant Enterobacteriaceae (CRE).

Antibiotic susceptibility testing was performed using a VITEK^®^ 2 system (bioMérieux, Inc., Hazelwood, MO, USA), with the results interpreted according to the existing guidelines [18]. Clinical and Laboratory Standards Institute (CLSI) recommendations and criteria for all bacteria cultured were used to define susceptibility to antimicrobial agents [19].

### 2.4. Statistical Analysis

Data analysis was performed using SPSS version 27. Descriptive statistics provided a summary of demographic and clinical characteristics. The proportions of microbial species and resistance patterns between the two groups were compared using the Chi-square test or the Fisher’s exact test, based on the frequency assumptions. For continuous data we calculated the independent samples *t*-test when comparing two means, and the Mann–Whitney U test to compare two medians. Logistic regression models were used to identify preterm birth risk factors independently associated with microbial complexity and resistance patterns. A *p*-value < 0.05 was considered statistically significant.

## 3. Results

Table 1 detailed the background characteristics of pregnant women with UTIs, distinguishing between preterm (*n* = 126) and full-term (*n* = 182) groups. The mean age for the preterm group was 27.5 years and 28.3 years for the full-term group, with no significant difference (*p*-value = 0.136). BMI categories showed a majority in the 25–29.9 kg/m^2^ range for both groups, with no significant difference (60.3% in the preterm group vs. 53.8% in the full-term group, *p*-value = 0.483). Smoking during pregnancy was reported by 9.5% of pregnant women with UTI who gave birth preterm, compared with 12.1% in the full-term group (*p*-value 0.480).

Regarding parity, 46.0% of the preterm group were primigravida, compared with 45.1% in the full-term group (*p*-value = 0.865). In terms of the trimester of infection, the third trimester saw 30.2% of infections in the preterm group and 29.7% in the full-term group, with no significant difference overall in trimester distributions (*p*-value = 0.932). The medical history aspect revealed that 44.4% of the preterm group had a previous UTI, compared with 38.5% in the full-term group. Despite this higher percentage in the preterm group, the difference was not statistically significant (*p*-value = 0.293).

Table 2 compares laboratory data and culture results between UTI patients who gave birth preterm and those who had full-term births. Significant differences were observed in several markers, indicating infection and inflammation. The median WBC count, an indicator of infection, was significantly higher in the preterm group (12.3) compared with the full-term group (9.1), with a *p*-value of 0.032. Similarly, neutrophils showed a significantly higher median count in the preterm group (7.8) compared with the full-term group (6.1), with a *p*-value of less than 0.001.

C-reactive protein also showed a notable difference. The preterm group had a significantly higher median CRP level (18) than the full-term group (7), indicating a heightened inflammatory response in preterm births (*p*-value < 0.001). Additionally, the distribution of bacteria in urine cultures was significantly different. A higher percentage of preterm patients had cultures with two or more bacteria (42.1%) compared with the full-term group (27.5%), with a *p*-value of 0.009. Other variables, including lymphocytes, platelets, red blood cells, hemoglobin, creatinine, and urea, did not show statistically significant differences, although some trends were noted.

Among the Gram-negative organisms, *Escherichia coli* was the most commonly identified in both preterm (65.9%) and full-term (58.8%) births, although the difference was not statistically significant (*p*-value = 0.208). *Klebsiella* spp. showed a significant difference; it was more prevalent in full-term births (21.4%) compared with preterm births (11.1%) with a *p*-value of 0.018. *Enterobacter* spp. was significantly more prevalent in preterm births (21.4%) than in full-term births (9.9%), with a *p*-value of 0.004. Other Gram-negative organisms, such as *Pseudomonas* spp., *Proteus* spp., and *Bacteroides* spp., did not show statistically significant differences between the two groups.

In the Gram-positive category, Group B *Streptococcus* (GBS) was notably more prevalent in preterm births (17.5%) compared with full-term births (1.6%), with a *p*-value of less than 0.001. Other Gram-positive bacteria like *Enterococcus* spp., *Streptococcus* spp., and *Staphylococcus* spp. did not show significant differences between preterm and full-term births, as presented in Table 3.

The findings reveal a significantly higher prevalence of extended-spectrum beta-lactamases (ESBL) producing organisms in the preterm group (19.8%) compared with the full-term group (4.4%), with a *p*-value of less than 0.001. Vancomycin-resistant Enterococci (VRE) were also more prevalent in the preterm group (4.0%) than in the full-term group (0.5%), with a *p*-value of 0.032. Similarly, carbapenem-resistant Enterobacteriaceae (CRE), a serious concern in antibiotic resistance, were found exclusively in the preterm group (4.8%) with a significant *p*-value of 0.003.

**Figure 1:**
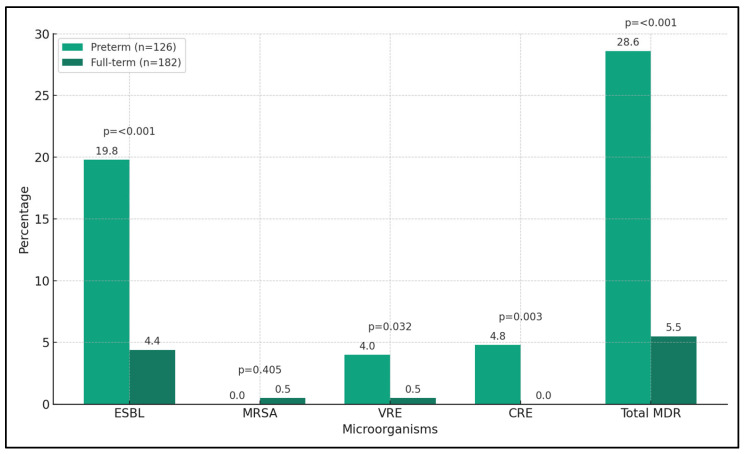
Evidence of MDR organisms isolated from urine cultures.

Methicillin-resistant Staphylococcus aureus (MRSA) was identified in one case in the full-term group (0.5%) and none in the preterm group, but the difference was not statistically significant (*p*-value = 0.405). When considering the total incidence of MDR organisms, the preterm group had a significantly higher rate (28.6%) compared with the full-term group (5.5%), with a *p*-value of less than 0.001, as seen in Table 4 and Figure 1.

A significant finding was the higher resistance to Amoxicillin in the preterm group (20.6%) compared with the full-term group (6.6%), with a *p*-value of less than 0.001. This suggests a strong association between amoxicillin resistance and preterm births in the context of UTIs. Similarly, resistance to second-generation cephalosporins was significantly higher in the preterm group (17.4%) versus the full-term group (5.5%), with a *p*-value of less than 0.001. Resistance to third-generation cephalosporins also followed this trend, with the preterm group showing a 19.8% resistance rate compared to 6.6% in the full-term group, with a *p*-value of less than 0.001.

Fosfomycin showed a notable difference in resistance patterns, with the preterm group having a higher resistance rate (14.3%) compared with the full-term group (4.9%), and a significant *p*-value of 0.004. For other antibiotics, including nitrofurantoin, ampicillin/sulbactam, macrolides, piperacillin/tazobactam, penems (meropenem/imipenem), glycopeptides, fourth-generation cephalosporins, ticarcillin/clavulanic, and piperacillin, the differences in resistance between the preterm and full-term groups were not statistically significant, as described in Table 5.

The analysis identified several significant risk factors associated with increased odds of preterm birth. Group B *Streptococcus* infection was found to be a substantial risk factor, with a coefficient (β) of 0.92 and an odds ratio of 2.5, indicating that the presence of GBS infection more than doubled the odds of preterm birth (*p* = 0.001). Similarly, infections caused by *Enterobacter* spp. showed a notable increase in preterm birth risk with an OR of 1.8 (*p* = 0.022). The presence of multidrug resistance was another significant predictor, with a high coefficient of 1.16 and an OR of 3.2, suggesting that MDR infections could triple the odds of preterm birth (*p* < 0.001). Extended-spectrum beta-lactamase (ESBL) production was also associated with increased risk, having an OR of 2.7 (*p* = 0.003).

**Figure 2:**
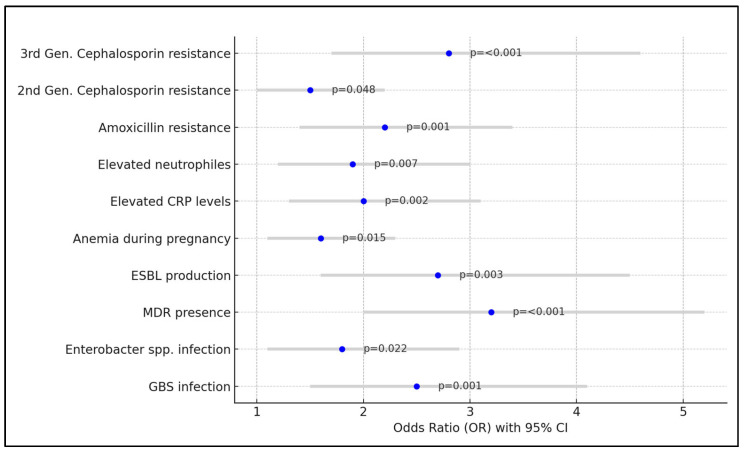
Risk factor analysis for preterm birth.

Other medical conditions during pregnancy, like anemia and elevated C-reactive protein (CRP) levels, were associated with increased odds of preterm birth, with ORs of 1.6 and 2.0, respectively. Elevated neutrophile count was similarly associated, with an OR of 1.9. Antibiotic resistance patterns also significantly impacted the risk, with amoxicillin resistance, second-generation cephalosporin resistance, and third-generation cephalosporin resistance having ORs of 2.2, 1.5, and 2.8, respectively, all indicating an increased risk of preterm birth, as presented in Table 6 and Figure 2.

## 4. Discussion

### 4.1. Literature Findings

The current study found significant differences in microbial species and multidrug resistance patterns between urinary tract infections (UTIs) in preterm and full-term births. Notably, the prevalence of Group B *Streptococcus* (GBS) and *Enterobacter* spp. was significantly higher in the preterm group, indicating a distinct bacterial profile associated with preterm births, in turn suggesting that these pathogens might contribute to the induction of inflammatory responses, potentially leading to preterm labor.

Furthermore, the study revealed a notable rise in multidrug-resistant organisms, particularly extended-spectrum beta-lactamases (ESBL) producing organisms and carbapenem-resistant Enterobacteriaceae (CRE) in the preterm birth group, highlighting the complex challenge of managing UTIs in pregnant women due to evolving resistance patterns. The increased resistance to antibiotics such as amoxicillin and cephalosporins in the preterm group underscored the urgency for personalized antimicrobial therapies informed by robust microbial identification and sensitivity testing.

These findings demonstrate a clear divergence in the microbial landscape and resistance patterns between the two groups. The significant differences observed emphasize the need for a nuanced understanding of the interplay between microbial pathogenesis, antibiotic resistance, and pregnancy outcomes, pointing to the importance of integrating detailed microbial analysis into clinical practice to better inform treatment choices for UTIs in pregnant women, particularly those at risk of preterm birth.

In an important study from California, it was demonstrated that urinary tract infections significantly increase the risk of preterm birth, particularly spontaneous preterm birth, with an adjusted risk score that is 1.4 times higher than the normal population [4]. This risk has been reported to persist across all trimesters; however, notably, women hospitalized with UTIs during their second trimester faced a threefold increased risk of delivering before 32 weeks. This finding is in line with previous studies [20,21] that have reported a two-fold increase in the odds of preterm birth for women with UTIs during pregnancy.

Contrasting findings have emerged regarding the association between pyelonephritis and preterm birth. While studies by Farkash et al. [22] and Bánhidy et al. [23] show an increased odds of preterm birth with pyelonephritis, Chen et al. did not find a significant association [24]. Another study confirmed an association between pyelonephritis and preterm birth, but the risk was not markedly higher than that associated with acute cystitis. This highlights in particular that hospitalization for UTIs during the second trimester significantly elevates the risk of preterm birth, in turn suggesting that severe infections during this period are particularly detrimental [25].

Considering that the current study used a monocentric database, it is important to acknowledge geographical and population-based differences in terms of UTIs. For example, one study from UAE found around 37% of pregnant symptomatic patients with UTI not exhibiting any growth of uropathogens in their urine [26], a proportion exceeding that reported in some other research [27]. However, in our study we excluded the negative samples to better determine antibiotic resistance patterns and MDR.

Similar to our study, *E. coli* is the most commonly isolated pathogen in different studies, but with relatively different prevalences, as low as 27% in Dube’s et al. study [26], compared with other studies [28,29], while the prevalence of GBS and *K. pneumoniae* is reported in higher prevalence in other research [28,30]. Even though we identified only 3.6% of *Staphylococcus* isolates, in the UAE study, no cultures identified *Staphylococcus* spp. [26,31]. Therefore, it can be assumed that the local microbial profile, including the predominance of *E. coli* in recurrent UTIs, likely reflects geographical and sociocultural influences.

In our study, the observed higher prevalence of ESBL resistance compared with other resistance types, such as MRSA, may be attributable to several factors. Primarily, the distribution and nature of the infecting organisms are crucial determinants. ESBL production is predominantly associated with Enterobacteriaceae, especially *Escherichia coli* and *Klebsiella* spp., which were significantly more prevalent in our cohort. Considering the global rise in ESBL-producing *E. coli* infections, our findings align with the broader trend of increasing ESBL resistance rates, potentially reflecting local or regional antibiotic prescribing patterns and subsequent selection pressures. In contrast, MRSA, which is associated with *Staphylococcus aureus*, had a very low incidence in our study population. This discrepancy could be due to several factors, including the natural prevalence of these bacteria in pregnant women, the specific susceptibility of these organisms to the antibiotics used in this population, and the overall lower rate of *S. aureus* as a causative agent of urinary tract infections compared with *E. coli.* Additionally, the zero incidence of MRSA in the preterm group versus the full-term group might not necessarily indicate a trend but could be a result of the smaller sample size or lower exposure to risk factors for MRSA in the preterm group.

Interestingly, antimicrobial resistance for *E. coli* was lower in other studies, compared with our findings of 10.7% ESBL organisms, while GBS showed higher sensitivity to common antibiotics [32,33]. Regarding antibiotic use, most studies favor nitrofurantoin as the first-line treatment for UTIs in pregnant women, except for specific conditions and late pregnancy [34]. High organism sensitivity to penicillins, erythromycin, and cephalosporins is noted in the literature, with moderate sensitivity to cefuroxime, commonly used in empirical therapy for culture-negative cases, comparable to an overall resistance percentage of 12.1% for second-generation cephalosporines in our study, although it was significantly higher among mothers who gave birth preterm [35].

Regarding the background data of participants, the association of demographic factors like age, BMI, and parity with UTI remains unclear due to conflicting evidence from various studies [36], the majority of pregnant women with UTI were in their second trimester, differing from another study where symptoms were more common in the first trimester [31]. Notably, diabetes and a previous history of UTI were identified as risk factors for UTI, in line with broader research [5]; however, the expected association with hypothyroidism was only observed in two symptomatic women [37], suggesting a need for further research to establish more definitive associations. When comparing culture-negative and culture-positive groups, the latter had significantly more known risk factors although individual comparisons were limited by small group sizes [26].

This study illuminates the critical need for personalized management of UTIs in pregnant women, particularly against a backdrop of increasing multidrug resistance. Our findings reveal distinct microbial profiles and resistance patterns in preterm versus full-term births, underscoring the importance of precise microbial identification and tailored antimicrobial therapies. The significant prevalence of multidrug-resistant organisms in preterm births further emphasizes the necessity for vigilant, adaptive treatment strategies and ongoing surveillance. These insights are pivotal for informing future clinical guidelines and research, aiming to enhance maternal and neonatal outcomes by effectively combating UTIs in pregnancy.

Furthermore, while this study posits a global applicability given the widespread prevalence of urinary tract infections, it is imperative to acknowledge the specific demographic nuances of the Romanian population from which the sample was drawn. Predominantly composed of white, middle-class Caucasian women, this homogeneity presents unique characteristics that may influence the generalization of the findings.

### 4.2. Study Limitations

The study’s methodological approach, while robust, presents several limitations. The case–control design conducted in a single clinical setting in western Romania may limit the generalizability of the findings to wider populations due to regional differences in microbial prevalence, antibiotic use, and resistance patterns. Furthermore, the retrospective nature of data collection from patient records introduces the potential for information bias, particularly when accurately capturing lifestyle factors and medical history, which are often reliant on patient self-reporting, especially in the context of self-medicating habits with over-the-counter antibiotics. The exclusion of certain high-risk pregnancy conditions, such as previous preterm births and genetic abnormalities, while strengthening the study’s focus, also restricts the applicability of findings to all pregnant women with UTIs. Additionally, while the study utilized comprehensive microbial identification and sensitivity testing, rapid changes in resistance patterns may necessitate continual updates to the data for sustained relevance. A limitation of this study is the unavailability of data on concurrent medications for conditions like hypertension and diabetes, which could impact UTI treatment outcomes. Moreover, data that can potentially influence the UTIs during pregnancy, such as recent travel and the sexual intercourse pattern were not available due to the retrospective nature of the study. Lastly, the study’s reliance on statistical significance might not fully capture the clinical importance of the observed differences, particularly in cases where statistical power is limited by sample size.

## 5. Conclusions

This study identified significant variances in the microbial flora and multidrug resistance patterns between UTIs in preterm and full-term births, highlighting the complex interplay between microbial resistance and pregnancy outcomes. The increased incidence of multidrug-resistant organisms, particularly ESBL producers and carbapenem-resistant Enterobacteriaceae in preterm births, underscores the urgency of developing targeted antimicrobial strategies. The association of specific bacteria, like Group B *Streptococcus* and *Enterobacter* spp., with preterm deliveries further emphasizes the need for precise microbial identification. Future research should focus on exploring novel antimicrobial agents and personalized treatment approaches, guided by in-depth microbial profiling and resistance pattern analysis, to improve the management of UTIs in pregnant women and reduce the risk of adverse birth outcomes.

## Figures and Tables

**Table 1:** Background characteristics of pregnant women presenting with UTI.

Variables	Preterm (*n* = 126)	Full Term (*n* = 182)	*p*-Value
Age (mean ± SD)	27.5 ± 4.2	28.3 ± 4.9	0.136
Age category			0.929
18–24 years	35 (27.8%)	48 (26.4%)	
25–29 years	42 (33.3%)	64 (35.2%)	
30–34 years	29 (23.0%)	45 (24.7%)	
≥35 years	20 (15.9%)	25 (13.7%)	
BMI *			0.483
<25 kg/m^2^	34 (27.0%)	54 (29.7%)	
25–29.9 kg/m^2^	76 (60.3%)	98 (53.8%)	
≥30 kg/m^2^	16 (12.7%)	30 (16.5%)	
Lifestyle			
Smoking during pregnancy	12 (9.5%)	22 (12.1%)	0.480
Alcohol use during pregnancy	8 (6.3%)	15 (8.2%)	0.544
Professional status			0.655
Employed	68 (54.0%)	104 (57.1%)	
Unemployed	20 (15.9%)	33 (18.1%)	
Student	15 (11.9%)	21 (11.5%)	
Housewife	23 (18.3%)	24 (13.2%)	
Parity			0.865
Primigravida	58 (46.0%)	82 (45.1%)	
Multigravida	68 (54.0%)	100 (54.9%)	
Trimester of infection			0.932
1st Trimester	40 (31.7%)	55 (30.2%)	
2nd Trimester	48 (38.1%)	73 (40.1%)	
3rd Trimester	38 (30.2%)	54 (29.7%)	
Medical history			
Previous UTI	56 (44.4%)	70 (38.5%)	0.293
Hypertension	20 (15.9%)	31 (17.0%)	0.787
Diabetes	18 (14.3%)	22 (12.1%)	0.572
Anemia	23 (18.3%)	29 (15.9%)	0.593
Asthma	10 (7.9%)	16 (8.8%)	0.790
Others	12 (9.5%)	20 (11.0%)	0.678
UTI symptoms			
Increased urinary frequency	67 (53.2%)	92 (50.5%)	0.650
Dysuria	58 (46.0%)	80 (44.0%)	0.718
Urgency	51 (40.5%)	71 (39.0%)	0.796
Hematuria	20 (15.9%)	26 (14.3%)	0.701
Cloudy/dark/strong-smelling urine	45 (35.7%)	61 (33.5%)	0.689
Suprapubic pain	18 (14.3%)	40 (22.0%)	0.089
Fever and chills	21 (16.7%)	19 (10.4%)	0.109
Back pain	23 (18.3%)	30 (16.5%)	0.685

*—adjusted to pregnancy; SD—standard deviation; UTI—urinary tract infection.

**Table 2:** Comparison of laboratory data and culture results between patients with a UTI who gave birth preterm and those with a UTI who gave birth full term.

Variables	Preterm (*n* = 126)	Full Term (*n* = 182)	*p*-Value
Blood tests (median, IQR)			
WBC (4.5–11.0 × 10^3^/mm^3^)	12.3 (10.5–14.7)	9.1 (7.4–11.2)	0.032
Lymphocytes (1.0–4.0 × 10^3^/mm^3^)	1.9 (1.4–2.5)	2.3 (1.8–2.9)	0.227
Neutrophils (1.5–8.0 × 10^3^/mm^3^)	7.8 (6.8–8.3)	6.1 (4.9–6.7)	<0.001
PLT (150–450 thousands/mm^3^)	283 (189–352)	326 (241–390)	0.055
RBC (3.9–5.5 × 10^6^/mm^3^)	4.2 (3.5–4.7)	4.6 (4.1–5.0)	0.308
Hemoglobin (12–16 g/L)	12.2 (10.5–13.0)	13.8 (12.9–14.2)	0.092
CRP (0–10 mg/L)	18 (14–26)	7 (4–10)	<0.001
Creatinine (0.6–1.1 mg/dL)	0.9 (0.7–1.3)	0.8 (0.6–1.0)	0.526
Urea (7–20 mg/dL)	17 (14–21)	12 (9–15)	0.060
Urine cultures (*n* = 308)			0.009
1 bacterium	73 (57.9%)	132 (72.5%)	
2 bacteria	33 (26.2%)	38 (20.9%)	
≥3 bacteria	20 (15.9%)	12 (6.6%)	

WBC—white blood cells; CRP—C-reactive protein; RBC—red blood cells; IQR—interquartile range; UTI—urinary tract infection; PLT—platelets; A *p*-value threshold of less than 0.05 was set for statistical significance.

**Table 3:** Type of organism isolated in urine cultures of pregnant women with a UTI, stratified by preterm and full-term births.

Microbial Identification	Preterm (*n* = 126)	Full Term (*n* = 182)	*p*-Value
Gram-negative			
*Escherichia coli* = 190/308 (61.7%)	83 (65.9%)	107 (58.8%)	0.208
*Klebsiella* spp. = 53/308 (17.2%)	14 (11.1%)	39 (21.4%)	0.018
*Pseudomonas* spp. = 38/308 (12.3%)	16 (12.7%)	22 (12.1%)	0.872
*Enterobacter* spp. = 45/308 (14.6%)	27 (21.4%)	18 (9.9%)	0.004
*Proteus* spp. = 23/308 (7.5%)	9 (7.1%)	14 (7.7%)	0.856
*Bacteroides* spp. = 18/308 (5.8%)	6 (4.8%)	12 (6.6%)	0.501
Gram-positive			
*Enterococcus* spp. = 23/308 (7.5%)	7 (5.6%)	16 (8.8%)	0.289
*Streptococcus* spp. = 22/308 (7.1%)	11 (8.7%)	11 (6.0%)	0.368
*Group B Streptococcus* = 25/308 (8.1%)	22 (17.5%)	3 (1.6%)	<0.001
*Staphylococcus* spp. = 11/308 (3.6%)	4 (3.2%)	7 (3.8%)	0.754

UTI—urinary tract infection.

**Table 4:** Evidence of multidrug resistant microorganisms isolated from urine cultures of patients with a UTI.

Urine Samples (*n* = 308)	Preterm (*n* = 126)	Full Term (*n* = 182)	*p*-Value
ESBL = 33 (10.7%)	25 (19.8%)	8 (4.4%)	<0.001
MRSA = 1 (0.3%)	0 (0%)	1 (0.5%)	0.405
VRE = 6 (1.9%)	5 (4.0%)	1 (0.5%)	0.032
CRE = 6 (1.9%)	6 (4.8%)	0 (0.0%)	0.003
Total MDR = 46 (14.9%)	36 (28.6%)	10 (5.5%)	<0.001

UTI—urinary tract infection; ESBL—extended-spectrum beta-lactamases; MRSA—methicillin-resistant Staphylococcus aureus; VRE—vancomycin-resistant Enterococci; CRE—carbapenem-resistant Enterobacteriaceae; MDR—multidrug resistant.

**Table 5:** Evaluation of antibiotic resistance patterns from urine cultures compared between patients with UTIs who gave birth preterm and patients with UTIs who gave birth full term.

Antibiotic Resistance *	Preterm (*n* = 126)	Full Term (*n* = 182)	*p*-Value
Amoxicillin = 36/294 (12.2%)	26 (20.6%)	12 (6.6%)	<0.001
Nitrofurantoin = 43/289 (14.9%)	15 (11.9%)	28 (15.4%)	0.386
Ampicillin/sulbactam = 25/206 (12.1%)	13 (10.3%)	12 (6.6%)	0.239
Macrolides = 20/148 (13.5%)	10 (7.9%)	10 (5.5%)	0.393
Fosfomycin = 27/145 (18.6%)	18 (14.3%)	9 (4.9%)	0.004
Piperacillin/tazobactam = 15/139 (10.8%)	8 (6.3%)	7 (3.8%)	0.315
Penems (meropenem/imipenem) = 19/166 (11.4%)	8 (6.3%)	11 (6.0%)	0.912
Glycopeptides = 18/201 (8.9%)	11 (8.7%)	7 (3.8%)	0.072
2nd Gen. cephalosporin = 32/265 (12.1%)	22 (17.4%)	10 (5.5%)	<0.001
3rd Gen. cephalosporin = 37/277 (13.4%)	25 (19.8%)	12 (6.6%)	<0.001
4th Gen. cephalosporin = 25/201 (12.4%)	5 (4.0%)	10 (5.5%)	0.540
Ticarcillin/clavulanic = 29/139 (20.9%)	7 (5.5%)	12 (6.6%)	0.709
Piperacillin = 11/108 (10.2%)	4 (3.2%)	7 (3.8%)	0.754

*—fluoroquinolones and aminoglycosides were not considered due to contraindications during pregnancy; UTI—urinary tract infection.

**Table 6:** Risk factor analysis for preterm birth in the context of UTI and multidrug resistance.

Significant Risk Factors	Coefficient (β)	SE	OR	95% CI	*p*-Value
GBS infection	0.92	0.30	2.5	1.5–4.1	0.001
*Enterobacter* spp. infection	0.59	0.26	1.8	1.1–2.9	0.022
MDR presence	1.16	0.32	3.2	2.0–5.2	<0.001
ESBL production	0.99	0.35	2.7	1.6–4.5	0.003
Anemia during pregnancy	0.47	0.21	1.6	1.1–2.3	0.015
Elevated CRP levels	0.69	0.24	2.0	1.3–3.1	0.002
Elevated neutrophils	0.64	0.28	1.9	1.2–3.0	0.007
Amoxicillin resistance	0.79	0.33	2.2	1.4–3.4	0.001
2nd Gen. cephalosporin resistance	0.41	0.20	1.5	1.0–2.2	0.048
3rd Gen. cephalosporin resistance	1.03	0.37	2.8	1.7–4.6	<0.001

OR—Odds Ratio; CI—Confidence Interval; MDR—Multidrug Resistant; ESBL—Extended-Spectrum Beta-Lactamases; GBS—Group B *Streptococcus*; UTI—Urinary Tract Infection; SE—Standard Error.

## Data Availability

Data are available on request.
